# Antimicrobial Nanoparticles Against Superbugs: Mechanistic Insights, Biomedical Applications, and Translational Frontiers

**DOI:** 10.3390/ph18081195

**Published:** 2025-08-13

**Authors:** Ayman Elbehiry, Adil Abalkhail

**Affiliations:** Department of Public Health, College of Applied Medical Sciences, Qassim University, P.O. Box 6666, Buraydah 51452, Saudi Arabia; ar.elbehiry@qu.edu.sa

**Keywords:** antimicrobial nanoparticles, multidrug-resistant infections, targeted drug delivery, biofilm-associated infections, public health, translational nanomedicine

## Abstract

The accelerating threat of antimicrobial resistance (AMR) demands transformative strategies that go beyond conventional antibiotic therapies. Nanoparticles (NPs) have emerged as versatile antimicrobial agents, offering a combination of physical, chemical, and immunological mechanisms to combat multidrug-resistant (MDR) pathogens. Their small size, surface tunability, and ability to disrupt microbial membranes, generate reactive oxygen species (ROS), and deliver antibiotics directly to infection sites position them as powerful tools for infection control. This narrative review explores the major classes, mechanisms of action, and biomedical applications of antimicrobial NPs—including their roles in wound healing, implant coatings, targeted drug delivery, inhalation-based therapies, and the treatment of intracellular infections. We also highlight the current landscape of clinical trials and evolving regulatory frameworks that govern the translation of these technologies into clinical practice. A distinctive feature of this review is its focus on the interplay between NPs and the human microbiota—an emerging frontier with significant implications for therapeutic efficacy and safety. Addressing this bidirectional interaction is essential for developing microbiota-informed, safe-by-design nanomedicines. Despite promising advances, challenges such as scalability, regulatory standardization, and long-term biosafety remain. With interdisciplinary collaboration and continued innovation, antimicrobial NPs could reshape the future of infectious disease treatment and help curb the growing tide of AMR.

## 1. Introduction

Antimicrobial resistance (AMR) poses a major global health challenge, undermining the efficacy of antibiotics that have long served as the cornerstone of modern medicine. Multidrug-resistant (MDR) pathogens—particularly those in the ESKAPE group, including *Enterococcus faecium* (*E. faecium*), *Staphylococcus aureus* (*S. aureus*), *Klebsiella pneumoniae K. pneumoniae*), *Acinetobacter baumannii* (*A. baumannii)*, *Pseudomonas aeruginosa* (*P. aeruginosa*), and *Enterobacter* species—are responsible for a significant proportion of hospital-acquired infections and exhibit resistance to many first-line treatments [1,2]. Global estimates indicate that AMR was directly responsible for 1.27 million deaths in 2019 and contributed to nearly 5 million fatalities overall [3]. Without effective intervention, AMR could result in 10 million deaths annually by 2050—surpassing cancer as the leading cause of mortality [4,5].

Exacerbating this crisis is the stagnation in antibiotic discovery, as most newly approved agents are structural derivatives of existing drug classes, offering limited innovation [6,7]. Treatment challenges are further compounded by infections involving biofilms and intracellular pathogens, which hinder the delivery and efficacy of conventional drugs [8,9,10]. These limitations underscore the urgent need for advanced antimicrobial platforms that not only exert direct bactericidal effects but also enhance targeted delivery, circumvent resistance mechanisms, and minimize host toxicity.

In response, nanoparticles (NPs) have emerged as promising antimicrobial agents. Their unique size-dependent properties, high surface area-to-volume ratio, and functional tunability enable multiple mechanisms of action [11,12]. Metallic NPs—such as silver (AgNPs), gold (AuNPs), zinc oxide (ZnO NPs), and copper oxide (CuO NPs)—exhibit broad-spectrum activity against multidrug-resistant (MDR) pathogens [13,14,15]. Their antimicrobial mechanisms include reactive oxygen species (ROS) generation, membrane disruption, DNA interaction, protein inactivation, and inhibition of biofilm formation [16].

Unlike conventional antibiotics, which typically target a single cellular pathway, NPs can disrupt membranes, interfere with intracellular signaling, and generate ROS simultaneously. This multimodal action makes it more difficult for pathogens to develop resistance through single-point mutations. In addition to their inherent antimicrobial activity, NP-based formulations are increasingly designed to improve pharmaceutical properties such as solubility, systemic circulation time, tissue-specific targeting, and sustained release. These features are especially valuable for treating persistent or localized infections—such as chronic wounds, implant-associated infections, and pulmonary diseases—where traditional antibiotics often fail to reach therapeutic levels. Moreover, multifunctional nanocarriers, including stimuli-responsive and theranostic systems, are being developed to integrate diagnostic imaging, controlled drug release, and immunomodulatory activity into a single therapeutic platform [17].

Despite the availability of various antibiotic classes, many formulations suffer from poor tissue penetration, rapid clearance, and nonspecific biodistribution—particularly in complex microenvironments like biofilms, intracellular niches, and hypoxic tissues. These pharmacokinetic limitations reduce therapeutic efficacy and facilitate the emergence of resistant strains due to sub-therapeutic exposure [18,19]. Furthermore, sub-therapeutic antibiotic exposure in these compartments contributes to the selection of resistant strains and exacerbates AMR spread [20]. To address these issues, advanced delivery systems—including liposomes, polymeric NPs, and ligand-targeted nanocarriers—have been developed to enhance tissue-specific accumulation, prolong circulation, and enable controlled or responsive drug release [21]. These technologies are particularly promising for treating chronic infections, reducing systemic toxicity, and overcoming barriers that compromise conventional therapy. Recent advances in nanomedicine also emphasize the use of biodegradable and biocompatible materials to support safer clinical translation and reduce long-term toxicity risks. Collectively, these innovations align with the goals of precision nanomedicine, where treatment can be tailored to patient-specific microbiota, immune profiles, and resistance patterns.

Progress in antimicrobial nanomaterials has led to innovative therapeutic approaches. Yathavan et al. [22] demonstrated that AgNPs embedded in silk elastin-like protein hydrogels effectively inhibited *S. aureus* and *P. aeruginosa* biofilms while promoting mucosal tissue regeneration. Alhosani et al. [23] developed ZnO–Ag nanocomposite coatings that exhibited strong antibiofilm activity against *P. aeruginosa* through ROS-mediated membrane damage. Additionally, AgNPs have shown synergistic effects when combined with conventional antibiotics. Kamer et al. [24] reported that biosynthesized AgNPs significantly enhanced the antibacterial activity of gentamicin, ceftazidime, and ciprofloxacin against MDR and XDR *P. aeruginosa*, reducing minimum inhibitory concentrations (MICs) by 2–10–fold and suppressing virulence gene expression.

A novel strategy by Szymczak et al. [25] involved conjugating lytic T7 bacteriophages with AgNPs, resulting in a “phage-armed” nanoplatform with superior antibiofilm efficacy against *Escherichia coli* (*E. coli*) compared to either agent alone. This formulation demonstrated low cytotoxicity in mammalian cells, supporting its translational potential. Similarly, Ahmad et al. [26] showed that green-synthesized AgNPs from *Phoenix dactylifera* extract suppressed *P. aeruginosa* biofilm formation and virulence factors—such as swarming motility and pyocyanin production—by interfering with quorum sensing (QS) pathways. These studies collectively highlight the multifaceted potential of antimicrobial NPs in overcoming drug resistance, eradicating biofilms, and maintaining host compatibility. In parallel, a 2025 study by Ahmad et al. [26] investigated green-synthesized AgNPs derived from *Phoenix dactylifera* extract and revealed that sub-inhibitory concentrations (MIC/5 to MIC/2) substantially suppressed *P. aeruginosa* biofilm formation and virulence factor expression, including swarming motility, protease activity, and pyocyanin production.

These effects were attributed to interference with QS signaling pathways. Mechanistically, such antimicrobial action is supported by numerous studies confirming that NPs—particularly Ag and ZnO NPs—generate ROS, which induce lipid peroxidation, disrupt bacterial membranes, and lead to cell death. Collectively, these findings underscore the multifaceted potential of antimicrobial NPs not only in overcoming conventional resistance mechanisms and eradicating biofilms but also in maintaining host biocompatibility, thereby advancing their translational value in infection-prone clinical environments.

Nonetheless, clinical adoption of NP-based antimicrobials remains constrained by regulatory, manufacturing, and safety challenges. Key barriers include the absence of standardized classification systems, inconsistent toxicological profiles, and limited data on long-term biosafety [27,28]. Regulatory approval is further complicated by the lack of harmonized testing protocols and variable definitions of nanomaterials across jurisdictions [29,30]. Additionally, scalability issues, complex designs, and batch-to-batch variability hinder clinical translation beyond preclinical stages [31,32]. Recognizing these gaps, global health organizations such as the WHO and Horizon Europe have prioritized nanotechnology in the fight against AMR, supporting dedicated funding for translational research and regulatory alignment.

While previous reviews have addressed the synthesis and applications of antimicrobial NPs, their interaction with the host microbiota remains underexplored. This review provides an integrated perspective, emphasizing not only the physicochemical mechanisms and biomedical applications of NPs but also their bidirectional relationship with the gut microbiota—a critical aspect influencing host immunity, therapeutic efficacy, and safety. By addressing this knowledge gap, we propose a framework for the development of microbiota-informed nanotherapeutics in the fight against resistant pathogens.

**Figure 1:**
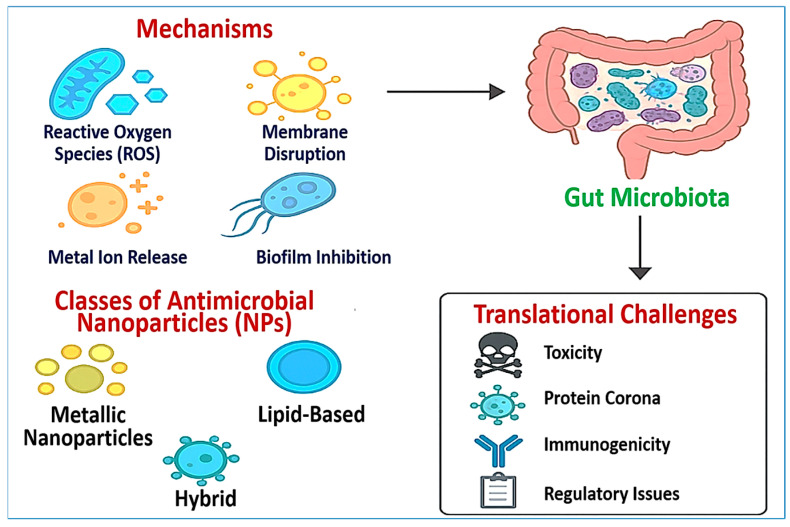
Schematic overview of antimicrobial NPs against superbugs, highlighting key mechanisms (ROS, membrane disruption, metal ion release, biofilm inhibition), major NP classes (metallic, lipid-based, hybrid), gut microbiota interactions, and translational challenges (toxicity, immunogenicity, protein corona, regulatory barriers).

From a pharmaceutical perspective, the formulation, scale-up, and pharmacokinetic optimization of NP-based antimicrobials must meet both therapeutic and regulatory requirements. This review synthesizes recent advances in NP types, antimicrobial mechanisms, biomedical applications, and translational barriers. To support this discussion, Figure 1 presents a conceptual overview of NP classes, core mechanisms, microbiota interactions, and major challenges impeding clinical adoption. Together, these insights underscore the need for safe, precise, and microbiota-conscious nanotherapeutic strategies to combat MDR infections.

## 2. Materials and Methods

This narrative review is based on a comprehensive literature search of peer-reviewed articles published between 2008 and 2025. The databases consulted included PubMed, Scopus, and Web of Science. The following search terms were used individually and in combination: “antimicrobial nanoparticles (NPs),” “nanomaterials,” “drug-resistant bacteria,” “biofilm inhibition,” “nanoparticle drug delivery,” “microbiota–NP interaction,” and “translational nanomedicine.” Preference was given to articles published in high-impact journals, systematic reviews, and original research studies with clinical or translational relevance. Only English-language publications were considered. Priority was placed on studies that described well-characterized nanoparticle systems, provided mechanistic insights into antimicrobial action, and included translational or toxicological evaluations. Articles were selected based on their relevance to the core themes of this review: the classes and mechanisms of antimicrobial NPs, biomedical applications, interactions with the gut microbiota, and translational or regulatory challenges. No new experimental data were generated or analyzed for this review.

## 3. Classes and Characteristics of Antimicrobial NPs

NPs designed for antimicrobial applications encompass a broad range of core materials, surface chemistries, and functional architectures. Critical physicochemical attributes—such as particle size, surface charge, morphology, and chemical composition—profoundly influence their antimicrobial potency, biocompatibility, biodistribution, degradation kinetics, and formulation feasibility for therapeutic deployment [12,13]. The antimicrobial efficacy of NPs arises from multifactorial and often synergistic mechanisms, including disruption of microbial membranes, generation of ROS, interference with protein synthesis and nucleic acid replication, and inhibition of QS and biofilm development [16,33]. These multifaceted actions confer a significant advantage over conventional antibiotics, particularly in combating MDR and biofilm-associated pathogens.

From a pharmaceutical development perspective, the rational design of antimicrobial NPs requires careful consideration of their physicochemical stability, site-specific targeting ability, controlled or sustained drug release profiles, and overall biocompatibility. Lipid-based nanocarriers, particularly liposomes, have advanced into clinical use due to their versatility in encapsulating both hydrophilic and lipophilic therapeutics, extending systemic circulation time, and minimizing off-target toxicity [34]. Similarly, solid lipid NPs and polymer-based systems offer enhanced physical stability and scalability for large-scale manufacturing [35]. This section integrates recent experimental studies that validate not only the antibacterial action of various NP classes but also their safety in mammalian cell models, aligning with translational goals.

### 3.1. Metal and Metal Oxide NPs

**Figure 2:**
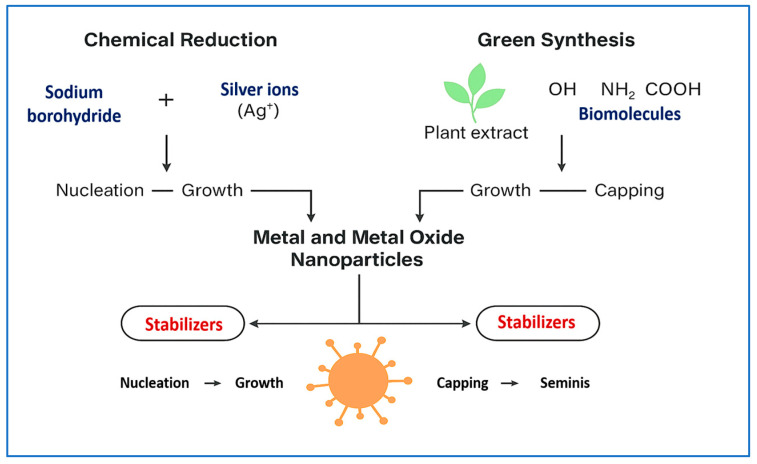
Schematic of chemical vs. green synthesis of metal/metal oxide NPs. Chemical routes use synthetic reducers (e.g., sodium borohydride), while green synthesis employs plant biomolecules for eco-friendly reduction and capping. Stabilizers (e.g., citrate, chitosan) prevent aggregation and tune surface properties.

This subsection not only outlines diverse synthesis strategies for metal-based NPs but also critically compares their effects on antimicrobial performance and translational potential. Metal-based NPs are foundational to antimicrobial nanotechnology due to their potent microbicidal activity, broad-spectrum efficacy, and versatility in biomedical applications [36,37]. Their antimicrobial properties stem from both intrinsic physicochemical features and synthesis-dependent characteristics. For instance, in classical chemical reduction methods, AgNPs are synthesized through the nucleation of silver ions (Ag^+^), which are reduced by agents such as sodium borohydride or plant-derived polyphenols [38]. This process initiates seed formation, followed by growth and capping steps. Stabilizers such as citrate, gelatin, or chitosan are then added to prevent agglomeration and to define the final particle size, shape, and surface chemistry [39]. A schematic overview of chemical and green synthesis pathways is presented in Figure 2, highlighting key stages—nucleation, growth, and capping—as well as the reagents and surface functionalization techniques involved.

Synthesis parameters, including reaction time, temperature, and the concentration of reducing agents, significantly influence critical properties such as particle size (typically 10–50 nm), zeta potential, and crystallinity. These parameters directly affect silver ion release and ROS generation, which are key determinants of antimicrobial activity [40]. Green synthesis approaches, which utilize phytochemicals and proteins, produce biofunctional surface groups (e.g., flavonoids, terpenoids, amines) that promote bacterial adhesion via hydrogen bonding or electrostatic interactions, thereby enhancing antimicrobial efficacy [41]. These structural and surface features govern membrane permeabilization, enzyme inactivation, and intracellular DNA disruption, all of which contribute to the observed variability in antimicrobial potency and therapeutic outcomes [42].

Recent experimental studies have validated the dual performance and safety of metal and metal oxide NPs. El-Fallal et al. [43] biosynthesized ZnO NPs using kombucha extract and demonstrated >90 % bactericidal activity against *E. coli* and *K. pneumoniae* at concentrations under 100 µg/mL. Tazeen et al. [44] designed chitosan–ZnO nanocomposites exhibiting potent antibiofilm efficacy and low cytotoxicity in human keratinocytes. These data confirm that optimized synthesis and surface engineering can yield metal-based nanomaterials that are both therapeutically potent and cytocompatible—supporting their translation into medical applications such as wound dressings, coatings, and topical antimicrobials.

In addition to established agents such as Ag, ZnO, CuO, and TiO_2_ NPs, platinum NPs (PtNPs) have emerged as a compelling class of antimicrobial nanomaterials due to their catalytic and redox-active properties. PtNPs exhibit broad-spectrum antibacterial activity—and in some formulations also antibiofilm action—through multiple mechanisms, including membrane disruption, ROS generation, and oxidative damage to intracellular biomolecules (e.g., DNA and proteins). Studies have shown that PtNPs maintain their antimicrobial efficacy in low-oxygen environments and synergize with conventional antibiotics to reduce bacterial growth and biofilm resilience [45,46,47,48,49]. Moreover, green synthesis approaches and surface functionalization strategies have improved their biocompatibility profiles, making PtNPs promising candidates for translational applications such as wound dressings, implant coatings, and targeted antimicrobial delivery.

AgNPs exhibit multifaceted antimicrobial mechanisms. These include the sustained release of Ag^+^, which disrupts thiol-containing enzymes and structural proteins, leading to enzymatic inhibition and loss of membrane integrity. Simultaneously, AgNPs induce oxidative stress through ROS generation, impairing bacterial DNA and protein synthesis and ultimately triggering microbial apoptosis [16,50]. Moreover, AgNPs interfere with QS pathways, thereby reducing bacterial virulence and inhibiting biofilm formation [51,52].

Recent advances in nanomedicine have facilitated the clinical translation of these mechanisms. For example, Chen et al. [53] developed a carrier-free injectable hydrogel incorporating AgNPs, which successfully eradicated methicillin-resistant *S. aureus* (MRSA) in a diabetic wound model. In addition to its antimicrobial efficacy, the nanocomposite promoted tissue regeneration and attenuated pro-inflammatory cytokine production, demonstrating dual functionality for infection control and wound healing.

ZnO NPs also display broad-spectrum antimicrobial activity, primarily mediated through ROS generation and zinc ion (Zn^2+^) release. Unlike AgNPs, ZnO NPs possess intrinsic photocatalytic properties under ultraviolet (UV) or visible light, further enhancing their disinfection capabilities. Gao et al. [54] reported several key mechanisms, including membrane destabilization, intracellular oxidative damage, and interference with QS and biofilm development. Translationally, Selmani et al. [55] fabricated ZnO–calcium carbonate nanocomposite coatings for titanium implants, achieving over 90% reduction in the viability of *S. aureus*, *S. epidermidis*, and *Candida albicans* (*C. albicans*), while preserving osteoblast compatibility—highlighting their promise in orthopedic applications.

CuO and titanium dioxide (TiO_2_) NPs also demonstrate strong antimicrobial properties, primarily via ROS-mediated microbial damage. CuO NPs release copper ions (Cu^2+^) and catalyze redox reactions that compromise bacterial membrane integrity and denature intracellular proteins and nucleic acids. These effects have been validated against MDR strains including *E. coli*, *K. pneumoniae*, *P. aeruginosa*, and MRSA, with MICs ranging from 62.5 to 125 µg/mL [56]. TiO_2_ NPs—particularly in their anatase crystalline form—act as photocatalysts capable of generating hydroxyl radicals and singlet oxygen under UV or visible light activation. A recent study showed that visible light-activated TiO_2_ nanostructures enhanced the antibacterial efficacy of conventional antibiotics against *P. aeruginosa* and *K. pneumoniae*, while maintaining low cytotoxicity [57]. However, the need for light activation and limited pharmacokinetic data remain barriers to systemic clinical use.

From a formulation perspective, the therapeutic performance and biosafety of metal and metal oxide NPs are closely tied to their physicochemical characteristics—such as particle size, morphology, surface charge, and colloidal stability. Optimizing these properties enhances microbial membrane targeting and minimizes off-target toxicity. Nonetheless, issues like nanoparticle aggregation in physiological environments often require stabilizing strategies such as PEGylation or surfactant-based coatings [56].

These NPs are increasingly incorporated into advanced delivery systems—including hydrogels, wound dressings, orthopedic cements, implant coatings, and sprayable formulations—offering localized and sustained antimicrobial effects while reducing systemic exposure. Despite promising preclinical results, clinical translation still demands comprehensive toxicological evaluation, scalable synthesis protocols, and harmonized regulatory standards [12]. Given their potent antimicrobial activity, modular design flexibility, and multi-targeted mechanisms, metal-based NPs remain at the forefront of next-generation antimicrobial strategies.

### 3.2. Polymeric NPs

Polymeric NPs represent a versatile and promising class of antimicrobial nanostructures, offering both intrinsic antimicrobial properties (in specific cases) and advanced capabilities for controlled drug delivery. These nanosystems are typically fabricated from biocompatible and biodegradable polymers such as chitosan, polylactic-co-glycolic acid (PLGA), and polyethylene glycol (PEG). Their physicochemical characteristics support prolonged drug release, site-specific targeting, and reduced systemic toxicity—attributes that are critical for therapeutic success and clinical translation [58,59].

Among polymeric NPs, chitosan-based formulations are particularly notable for their inherent antimicrobial activity, which arises from chitosan’s cationic nature. This allows for electrostatic interactions with the negatively charged bacterial membrane, leading to membrane destabilization, increased permeability, and cell death. Tazeen et al. [44] demonstrated that chitosan–ZnO nanocomposites not only eradicated *E. coli* and *P. aeruginosa* biofilms but also exhibited excellent cytocompatibility in human keratinocyte assays, underscoring their clinical potential.

PLGA-based NPs, approved by regulatory authorities such as the U.S. FDA and EMA, are among the most investigated nanocarriers for antimicrobial therapy. Their degradation kinetics can be fine-tuned by altering the lactic/glycolic acid ratio, allowing precise control over drug release profiles. Türeli et al. [59] reported that ciprofloxacin-loaded PLGA nanoparticles significantly enhanced pulmonary delivery and biofilm penetration in *P. aeruginosa*-infected airway models, with negligible cytotoxicity to CFBE41o^−^ and Calu-3 lung epithelial cells. Shariati et al. [58] similarly encapsulated tobramycin in PEG–PLGA nanoparticles and found that the formulation exhibited enhanced antibiofilm efficacy in artificial sputum while maintaining high cell viability in bronchial epithelial cultures.

PEGylation further improves the physicochemical stability and in vivo behavior of polymeric NPs. By reducing opsonization and immune clearance, PEG–PLGA nanoparticles prolong systemic circulation time and improve tissue penetration. Tchatchiashvili et al. [60] developed PEG–PLGA carriers targeting *P. aeruginosa* and *Burkholderia cenocepacia*, demonstrating both antimicrobial efficacy and epithelial cell compatibility, thus reinforcing their translational suitability for respiratory infections.

Moreover, polymeric nanocarriers can be engineered with stimuli-responsive elements or targeting ligands to achieve site-specific drug release. These smart systems can respond to pH gradients, enzymatic activity (such as bacterial enzymes), and redox changes typically present in infected microenvironments—thereby enhancing therapeutic precision and minimizing off-target effects. For example, Cöksu et al. [61] developed PLGA NPs loaded with antimicrobial peptides and decorated with enzyme-cleavable linkers to achieve selective release in *S. aureus*-infected tissues, showing enhanced antibacterial efficacy in vitro and in vivo with minimal mammalian cytotoxicity. Similarly, Miranda Calderón et al. [62] formulated antibody-functionalized polymeric NPs that selectively target bacterial cells, leveraging ligand-mediated specificity to improve antimicrobial action while preserving host cell viability.

Although specific glutathione (redox)-responsive antibiotic release systems are still emerging in microbial infection models, broad evidence supports the following principle: stimuli-sensitive polymeric nanocarriers enable environment-triggered drug release, thereby increasing efficacy and avoiding sub-therapeutic exposure, which promotes resistance [63]. In summary, polymeric NPs offer adaptable delivery platforms across administration routes—including intravenous, pulmonary, transdermal, and mucosal—making them a cornerstone in next-generation antimicrobial nanotherapies.

### 3.3. Lipid-Based NPs

Lipid-based NPs—including liposomes, solid lipid NPs, and nanostructured lipid carriers (NLCs)—have emerged as promising vehicles for antimicrobial delivery due to their excellent biocompatibility, drug encapsulation versatility, and sustained release potential. Constructed from physiologically compatible lipids, these systems are inherently suited for both systemic and localized administration, offering favorable safety profiles and low immunogenicity. Liposomes, composed of phospholipid bilayers encapsulating aqueous cores, are extensively studied for antibiotic and antimicrobial peptide delivery due to their membrane-mimetic properties, which facilitate fusion with microbial cells and intracellular delivery. Afrasiabi et al. [64] developed a doxycycline-loaded liposome doped with curcumin (NL-Cur + Dox) for combination antibacterial photodynamic therapy against *Aggregatibacter actinomycetemcomitans*. Their formulation achieved an 82.7 % reduction in biofilm mass and 75 % decrease in metabolic activity, with no significant cytotoxicity to human gingival fibroblasts (cell viability remained at ~ 90%, *p* = 0.074), as well as negligible hemolysis (< 5%), under therapeutic conditions.

SLNs and NLCs, considered next-generation lipid carriers, provide enhanced stability and drug loading relative to conventional liposomes. Arabestani et al. [65] reported that cationic SLNs attached more rapidly to bacterial cells and delivered superior antibacterial outcomes compared to their non-ionic counterparts, aligning with improved interactions and efficacy in wound infection models. Motsoene et al. [66] investigated multifunctional lipid-based nanoparticles in wound healing applications and found that these formulations supported sustained antimicrobial release, accelerated skin regeneration, and maintained fibroblast viability above 85 %—highlighting the translational synergy between infection control and tissue repair. Overall, lipid-based nanocarriers overcome limitations of traditional antimicrobials, particularly in biofilm-associated infections and delivery of hydrophobic agents. Their utility spans pharmaceutical, food, and cosmetic domains. However, rigorous in vivo and toxicological evaluations remain necessary to support regulatory approval and further clinical translation.

### 3.4. Hybrid NPs (MOF-Based Nanozymes)

Hybrid nanoparticles, particularly those incorporating metal–organic frameworks (MOFs) with catalytic or enzymatic properties, represent an innovative and multifunctional approach in antimicrobial nanotherapy [67,68]. These systems integrate multiple bactericidal mechanisms—such as metal ion release, ROS generation, and enzyme-mimetic activity—into a single nanosystem, enabling broad-spectrum antimicrobial efficacy while maintaining low cytotoxicity to host tissues.

For instance, Omer et al. [69] developed polycaprolactone–lipid hybrid NPs encapsulating ciprofloxacin, which provided sustained antibiotic release over seven days and significantly enhanced antibacterial activity against *E. coli* compared to free drug formulations [70]. This design demonstrated the ability of hybrid NPs to improve drug retention and therapeutic outcomes.

A notable example includes molybdenum-doped zeolitic imidazolate framework-8 nanozymes (Mo@ZIF-8), which possess intrinsic peroxidase-like activity. In the presence of trace hydrogen peroxide, Mo@ZIF-8 catalyzes the generation of hydroxyl radicals, producing potent bactericidal effects. Lian et al. [71] reported ≥ 99 % reduction in the viability of *E. coli* and *S. aureus* at concentrations as low as 10 µg/mL, with negligible cytotoxicity to mammalian cells.

Similarly, Shen et al. [72] designed a silk fibroin–lysozyme/ZIF-8 composite coating for titanium implants that combined enzymatic degradation of bacterial cell walls with controlled Zn^2+^ ion release. The coating achieved over 90% bacterial inhibition against *S. aureus* and *E. coli* in vitro and promoted anti-inflammatory macrophage polarization, osteogenic differentiation, and neovascularization in murine bone defect models—underscoring its dual function in infection control and tissue regeneration.

In another example, Cai et al. [73] engineered pH-responsive Fe_3_O_4_@ZIF-8 magnetic NPs loaded with norfloxacin, which exhibited enhanced antimicrobial efficacy under acidic conditions typical of infected sites. The system also demonstrated excellent biocompatibility, supporting its suitability for localized drug delivery. Additionally, Wang et al. [74] developed a glucose-responsive hollow molybdenum-based nanozyme (HMMo/GOx@P) that converts endogenous glucose into hydrogen peroxide, triggering hydroxyl radical formation for effective bacterial eradication. This platform successfully eliminated *E. coli* and *S. aureus* in diabetic wound models, accelerated tissue regeneration, and showed low systemic toxicity.

These MOF-based nanozymes exemplify the potential of hybrid platforms to integrate multiple antimicrobial strategies within a single therapeutic system. However, key challenges remain, including synthesis scalability, batch-to-batch reproducibility, regulatory compliance, and comprehensive biocompatibility assessments for clinical translation. To support comparative analysis, Table 1 summarizes recent studies describing the synthesis, structural features, and antimicrobial applications of metal and metal oxide NPs. Key parameters include synthesis methods, particle size, surface area, synthesis conditions, morphology, and target pathogens or biofilm activity—offering a broad overview of design strategies and translational relevance. This comparative table enhances clarity and complements the diversity of the nanomaterials discussed in Section 3.1, Section 3.2, Section 3.3 and Section 3.4. All entries are drawn from recent peer-reviewed studies to ensure scientific accuracy and current relevance.

## 4. Mechanisms of Antimicrobial Action of NPs

NPs exhibit potent antimicrobial activity through a variety of synergistic and multifactorial mechanisms [95]. Unlike conventional antibiotics, which typically target a single biochemical pathway, NPs can simultaneously disrupt multiple microbial processes, thereby reducing the likelihood of resistance development [96,97]. These mechanisms include membrane disruption, generation of ROS, release of antimicrobial metal ions, interference with protein and nucleic acid synthesis, inhibition of biofilm formation, suppression of QS, and modulation of host immune responses. The physicochemical properties of NPs—such as particle size, shape, surface charge, and chemical composition—play a critical role in mediating their interactions with microbial cells [12].

A primary mode of action involves the disruption of bacterial cell membranes. Metallic NPs such as AgNPs, ZnO NPs, and CuO NPs exhibit strong affinity for the negatively charged bacterial surface, where they adhere and penetrate the lipid bilayer. This results in increased membrane permeability, leakage of cellular contents, membrane depolarization, and eventual cell lysis [13,15]. Chitosan-based polymeric NPs also disrupt microbial membranes through their polycationic nature, destabilizing the envelope without the need for metal ion release [98].

Another key mechanism is the intracellular generation of ROS, including hydroxyl radicals, superoxide anions, and hydrogen peroxide. These reactive species damage essential biomolecules such as lipids, proteins, and DNA, leading to oxidative stress and cell death [14]. AgNPs and ZnO NPs are particularly known for their ROS-generating capabilities. Additionally, TiO_2_ NPs exhibit photocatalytic activity that enhances ROS production upon exposure to UV or visible light, making them suitable for environmental disinfection and sterilization of medical devices [54,57].

Controlled metal ion release also contributes to the antimicrobial activity of NPs. For instance, Ag^+^ ions released from AgNPs interact with thiol groups in enzymes and structural proteins, disrupting key metabolic pathways. These ions can also impair DNA replication and transcription, leading to genotoxic stress [15,50]. Similarly, Zn^2+^ and Cu^2+^ ions exhibit strong bactericidal effects by binding to bacterial proteins, nucleic acids, and components of the cell envelope [12].

Biofilm inhibition is another critical mechanism by which NPs enhance infection control. Biofilms protect bacteria from antibiotics and immune responses by forming a dense extracellular polymeric matrix. AgNPs and ZnO NPs can prevent bacterial adhesion, degrade biofilm matrix components, and reduce the viability of embedded bacterial populations [26]. Additionally, NPs can interfere with QS, the bacterial communication system that regulates biofilm maturation and virulence. For example, biosynthesized AgNPs derived from *Lactobacillus rhamnosus* significantly downregulated QS-related genes responsible for swarming motility, protease production, and biofilm formation in *P. aeruginosa* [51,52].

Certain NPs also penetrate host cells, enabling intracellular antimicrobial activity and immune modulation. This is especially valuable for treating infections caused by intracellular pathogens or those associated with immune evasion. Mesoporous silica nanoparticles (MSNs), for example, are efficiently internalized by antigen-presenting cells such as macrophages and dendritic cells. Within endosomal compartments, MSNs deliver their therapeutic cargo while exhibiting minimal cytotoxicity. Functionalized MSNs have been shown to stimulate immune responses by enhancing cytokine release and antigen presentation, making them promising candidates for both antimicrobial therapy and vaccine adjuvant development [99].

In parallel, manganese–phosphorus nanosheets have demonstrated the ability to augment humoral immunity in implant-associated infections. These inorganic nanostructures promote macrophage polarization toward the M1 phenotype and enhance dendritic cell maturation, leading to robust memory B-cell responses and antibody production. Such immunomodulatory effects contribute to durable protection against biofilm-forming pathogens commonly linked to implanted medical devices [100].

**Figure 3:**
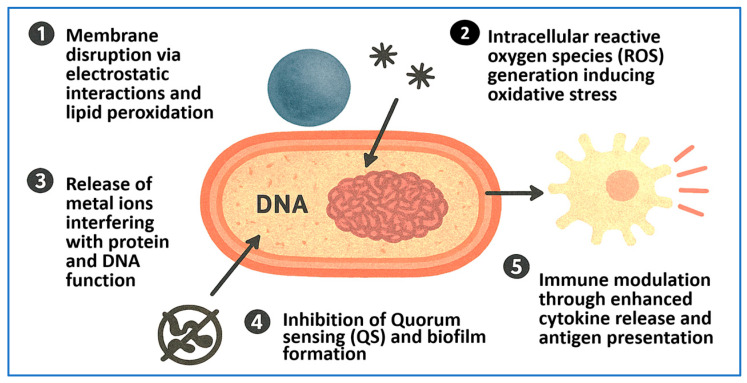
Mechanisms of antimicrobial action of NPs. This schematic illustrates the multifaceted bactericidal actions of NPs, including membrane disruption, ROS generation, metal ion release, quorum sensing inhibition, and immune modulation.

Figure 3 provides an integrated schematic summarizing the principal mechanisms of action of antimicrobial NPs. These include (1) microbial membrane disruption, (2) intracellular oxidative stress via ROS production, (3) metal ion release impairing enzyme function and DNA integrity, (4) inhibition of biofilm development and QS, and (5) immune activation through cytokine secretion, macrophage polarization, and antigen presentation. This multimodal strategy not only enhances pathogen clearance but also lowers the risk of resistance emergence.

To facilitate a comparative understanding of antimicrobial mechanisms, Table 2 summarizes the key features of various metal and metal oxide NPs—including their antimicrobial spectrum, MIC ranges, and cytotoxicity profiles. The data, derived from recent peer-reviewed studies, offer translational insights into their efficacy and biosafety, supporting informed decisions for clinical and biomedical applications.

## 5. Biomedical Applications of Antimicrobial NPs

The integration of antimicrobial nanoparticles (NPs) in biomedical applications has emerged as a transformative strategy to combat both persistent and emerging infectious diseases. This is especially relevant in the context of challenges posed by AMR, biofilm-associated infections, and intracellular bacterial persistence [120,121,122]. NPs offer several advantages over conventional antimicrobials, including their physicochemical tunability, high surface area-to-volume ratio, and the ability to be functionalized with both antimicrobial agents and targeting ligands. These properties enable site-specific drug delivery, controlled or sustained release, and multifaceted antimicrobial mechanisms—such as membrane disruption, ROS generation, and immune modulation. As a result, antimicrobial NPs are being increasingly employed across a broad range of biomedical applications, including wound dressings, implant coatings, targeted drug delivery systems, inhalation therapies, and immuno-engineered infection control strategies.

### 5.1. Wound Healing and Skin Infections

Wound management remains a significant challenge in infection control, particularly in chronic, non-healing wounds such as diabetic ulcers, pressure sores, and burn injuries [123]. These wounds are frequently colonized by MDR organisms such as *S. aureus* and *P. aeruginosa*, which delay tissue regeneration and increase the risk of systemic infections [124,125]. Antimicrobial NPs, especially AgNPs, have demonstrated strong efficacy in reducing microbial load while simultaneously promoting wound healing [126,127]. When incorporated into hydrogels, foams, or nanofibrous scaffolds, AgNPs release Ag^+^ ions that disrupt bacterial membranes, generate ROS, and downregulate virulence gene expression [128].

While AgNPs are well established for their antimicrobial and anti-inflammatory properties, AuNPs have recently gained attention as multifunctional agents in regenerative medicine. In a 2024 study, curcumin-loaded AuNPs (~ 42 nm) embedded in a Pluronic^®^ F127 hydrogel significantly accelerated wound closure in diabetic rat models. These NPs enhanced collagen deposition, stimulated angiogenesis, and reduced oxidative stress—outperforming free curcumin through sustained release and improved tissue penetration [129].

Additional studies have reported that green-synthesized AuNPs stabilized with biomolecules such as collagen or chitosan enhance fibroblast proliferation, re-epithelialization, and tissue remodeling. For example, collagen-coated AuNPs promoted dermal regeneration by lowering inflammatory cytokines (e.g., IL-6 and TNF-α) and upregulating growth factors such as basic fibroblast growth factor (bFGF) and vascular endothelial growth factor (VEGF) in human skin fibroblast cultures [130]. Moreover, VEGF-functionalized AuNPs have been shown to stimulate angiogenesis and accelerate healing in full-thickness skin wounds by modulating endothelial and fibroblast activity [131].

These findings underscore the potential of AuNP-based scaffolds and hydrogels as multifunctional platforms for wound healing—capable of integrating antimicrobial action, tissue regeneration, and controlled drug delivery. As hybrid nanomaterials continue to evolve by incorporating antibiotics, bioactive molecules, and anti-inflammatory agents, they are poised to revolutionize the management of chronic wounds.

### 5.2. Implant-Associated and Nosocomial Infections

Implantable medical devices—including catheters, orthopedic prostheses, and dental implants—are highly susceptible to colonization by biofilm-forming bacteria, which exhibit tolerance to systemic antibiotics and can evade host immune responses [132,133]. Once established, biofilms on implant surfaces often result in persistent infections that are difficult to eradicate and may necessitate device removal [134]. In response, nanoparticle-based surface modifications have emerged as a promising strategy to provide durable antimicrobial protection while preserving implant biocompatibility and mechanical integrity.

Among the most studied materials, ZnO and CuO NPs have demonstrated effective integration into implant coatings. These NPs release metal ions and generate ROS, producing bactericidal effects while simultaneously disrupting QS and preventing microbial adhesion. Alhosani et al. [23] reported that ZnO–Ag nanocomposite coatings successfully prevented *P. aeruginosa* biofilm formation and disrupted existing biofilms through both chemical (ROS generation) and physical (nanoscale sharpness) mechanisms.

Beyond metal oxides, gold nanostar (GNS) coatings have attracted interest for their dual role in antimicrobial photothermal therapy and promotion of osseointegration. Li et al. [135] demonstrated that titanium implants coated with GNSs and activated by near-infrared light achieved complete eradication of *S. aureus* while significantly enhancing osteoblast differentiation and bone regeneration in vivo. Similarly, gold-pre-deposited, ceramic-treated titanium showed antimicrobial activity against *Fusobacterium nucleatum* and *S. aureus* while maintaining corrosion resistance, making it suitable for dental applications [136].

Hybrid coatings combining NPs with antimicrobial peptides have also shown synergistic antimicrobial effects with reduced host cytotoxicity. For example, ZnO–CuS/F127 hydrogel coatings exhibited multienzyme-mimicking properties, significantly reduced biofilm biomass, and supported tissue regeneration without eliciting inflammatory responses [137].

Recent advances in nanostructured platforms have introduced multifunctional coatings that integrate graphene-based materials, chitosan, and AgNPs onto titanium surfaces. San et al. [138] developed reduced graphene oxide (rGO)/AgNP coatings using plasma electrolytic oxidation on 3D-printed porous titanium implants. These coatings eradicated over 99 % of *S. aureus* and MRSA biofilms while supporting osteoblast adhesion and proliferation—demonstrating both antimicrobial and osteoinductive potential.

Titanium nitride–silver (TiN–Ag) composite coatings have also demonstrated robust resistance to both Gram-positive and Gram-negative bacteria. Hojda et al. [139] showed that electrophoretically deposited TiN/Ag films significantly inhibited biofilm formation by *E. coli*, *S. aureus*, *E. faecalis*, and *E. faecium* while preserving critical implant properties such as hardness and corrosion resistance.

These innovative coatings underscore the translational potential of antimicrobial NPs in preventing implant-associated infections. By integrating potent bactericidal activity, biofilm inhibition, and host biocompatibility into durable surface materials, these nanostructures address one of the most persistent challenges in surgical and clinical settings. Notably, implant-coating formulations—including ZnO–Ag nanocomposites, Au nanostars, and graphene-based systems—have consistently demonstrated robust antibiofilm efficacy while preserving cytocompatibility with osteoblasts and epithelial cells, reinforcing their suitability for clinical translation.

### 5.3. Targeted Antibiotic Delivery Systems

NP-based antibiotic delivery platforms have revolutionized antimicrobial therapy by enhancing drug solubility, stability, and bioavailability while facilitating targeted delivery to sites of infection. These systems help overcome several limitations of conventional antibiotics that can lead to off-target toxicity, including poor tissue penetration, rapid degradation, and nonspecific distribution. By concentrating antimicrobial agents at the infection site and shielding them from inactivation, NPs offer a means of restoring antibiotic efficacy, particularly against MDR and persistent infections [97,140].

Lipid-based nanocarriers—including liposomes, solid lipid NPs, and NLCs—have been extensively used to encapsulate antibiotics such as rifampicin, ciprofloxacin, and vancomycin. These carriers extend systemic circulation times, enhance tissue accumulation, and reduce the systemic toxicity of their payloads. For example, maleimide-conjugated PEGylated liposomes have been engineered to deliver a wide range of β-lactam and aminoglycoside antibiotics, including ceftriaxone, doxycycline, cephalexin, and ampicillin. Ladva et al. [141] demonstrated that these liposomes significantly enhanced antibiotic uptake by bacterial cells, reduced the MICs by up to ninefold against *E. coli* and *K. pneumoniae*, and accelerated wound healing in vitro—all while maintaining low cytotoxicity.

Polymeric NPs, particularly those formulated from biodegradable and biocompatible polymers like PLGA and PEG, enable sustained and controlled antibiotic release [142,143]. These systems can be surface-functionalized with targeting moieties, such as folate or mannose, to direct drug-loaded NPs to infected cells, especially macrophages and dendritic cells. In a notable study, Maurya et al. [144] reported that folate–chitosan–decanoic acid-coated mesoporous silica NPs loaded with ethionamide effectively targeted alveolar macrophages in a *Mycobacterium tuberculosis* (*M. tuberculosis*) model. This approach enhanced intracellular drug concentrations and antimycobacterial activity over five days compared to non-targeted formulations.

Targeted delivery systems offer multiple clinical benefits. First, they minimize the required therapeutic dose by localizing antibiotic release at the site of infection, thereby reducing systemic toxicity and adverse effects. Second, they shield antibiotics from enzymatic degradation and efflux pumps—restoring efficacy against resistant pathogens. Third, ligand-mediated uptake by immune cells enables efficient treatment of intracellular infections caused by *M. tuberculosis*, *S. enterica*, and other intracellular pathogens.

Various nanocarriers—including liposomes, polymeric nanoparticles, dendrimers, and mesoporous silica NPs—are being actively optimized for translational antimicrobial applications. As these platforms continue to evolve, they offer considerable promise for precision therapy tailored to the infection site, pathogen profile, and resistance mechanism [145,146]. Importantly, both polymeric and lipid-based nanocarriers have demonstrated low cytotoxicity in mammalian cell models, with effective antimicrobial activity observed at concentrations well below cytotoxic thresholds—reinforcing their safety and clinical potential.

### 5.4. Inhalation-Based Therapies for Respiratory Infections

The respiratory tract is a major site of MDR infections, including hospital-acquired pneumonia, ventilator-associated pneumonia, and chronic respiratory conditions such as cystic fibrosis and bronchiectasis [147,148]. These infections present substantial therapeutic challenges due to biofilm formation, limited antibiotic penetration, and systemic toxicity associated with high-dose parenteral therapies. Inhalable NP-based delivery systems offer a promising strategy to overcome these limitations by providing localized, high-concentration drug delivery directly to the lungs while minimizing systemic exposure [149,150].

AgNPs have been widely investigated for inhalation therapy due to their broad-spectrum antimicrobial activity and immunomodulatory properties. In a murine model of *K. pneumoniae* pneumonia, aerosolized AgNPs significantly reduced bacterial loads in lung tissue, attenuated pro-inflammatory cytokine levels, and improved survival outcomes [151]. These findings underscore the potential of AgNPs to serve dual roles as antimicrobial and anti-inflammatory agents in the treatment of resistant pulmonary infections.

Beyond metallic NPs, polymer-based systems have also shown promise. Jalal et al. [152] developed methacrylated chitosan-coated ciprofloxacin nanoparticles designed for pulmonary delivery. These mucoadhesive particles adhered effectively to the lung mucosa and provided sustained drug release. In rat models of bronchopneumonia, the formulation enhanced antibiotic retention in the lungs and significantly improved bacterial clearance compared to conventional ciprofloxacin treatment. The strong mucoadhesive properties of the NPs also mitigated mucociliary clearance, which often limits the efficacy of inhaled drugs.

Liposomal formulations are another prominent class of inhalable nanocarriers. ARD-3150, a dry-powder liposomal ciprofloxacin formulation, has undergone evaluation in two Phase III clinical trials—ORBIT-3 and ORBIT-4. In patients with non-cystic fibrosis bronchiectasis chronically infected with *P. aeruginosa*, inhaled ARD-3150 significantly delayed the time to first pulmonary exacerbation and improved respiratory symptoms, confirming both the safety and therapeutic benefit of this platform.

Recent reviews have highlighted key advances in lipidic and polymeric nanocarriers tailored for pulmonary drug delivery. Lipid-based NPs enhance penetration through mucus and epithelial barriers and are compatible with inhalation devices such as nebulizers and dry-powder inhalers [153]. Concurrently, chitosan-based NPs offer robust mucoadhesion, enhanced drug stability, and extended lung retention—making them particularly suitable for managing chronic respiratory infections [154].

Inhalable NP systems enable targeted pulmonary delivery, extended therapeutic residence time, and reduced systemic exposure—establishing them as promising platforms for precision treatment of MDR respiratory infections. Preclinical inhalation studies consistently demonstrate high local antimicrobial efficacy with minimal off-target toxicity. Moreover, cytotoxicity assays confirm that these formulations preserve lung epithelial cell integrity, underscoring their potential for safe and effective clinical application in respiratory infection therapy.

### 5.5. Intracellular Infections and NP-Mediated Immunotherapy

Intracellular bacterial pathogens—such as *M. tuberculosis*, *S. enterica*, and *L. monocytogenes*—pose significant therapeutic challenges due to their ability to survive and replicate within host cells, effectively evading both immune surveillance and conventional antibiotics [146,155]. Many standard antimicrobials lack sufficient cell membrane permeability or are rapidly degraded in intracellular compartments, leading to therapeutic failure and persistent infection. NPs have emerged as powerful tools for overcoming these barriers, offering the capacity to deliver antimicrobial agents directly into infected cells and modulate host immune responses for improved treatment outcomes.

Surface-engineered NPs can be functionalized with targeting ligands that promote active uptake by phagocytic cells. For instance, Bera et al. [156] developed mannose-decorated solid lipid nanoparticles loaded with rifampicin to selectively target alveolar macrophages via mannose receptor-mediated endocytosis. These NPs achieved sustained intracellular drug release, significantly enhanced clearance of *Mycobacterium intracellulare*, and demonstrated minimal cytotoxicity to host cells—highlighting their potential for treating intracellular mycobacterial infections.

In a complementary approach, Dai et al. [157] designed an ROS-responsive nanosystem composed of vancomycin-loaded NPs cloaked in membranes derived from *Staphylococcus aureus*-infected macrophages. This biomimetic platform enabled targeted delivery to infection sites and triggered drug release in response to elevated ROS levels. The system exhibited potent bactericidal activity and accelerated wound healing in murine models, illustrating the therapeutic value of infection-responsive and immune-camouflaged nanocarriers.

These advanced platforms exemplify a dual-action therapeutic paradigm: facilitating the intracellular delivery of antibiotics while responding to infection-specific cues such as pH shifts and ROS accumulation for controlled drug release. Moreover, certain NPs can enhance host immune responses by promoting antigen presentation and stimulating cytokine production. For example, mesoporous silica NPs and manganese-based nanosheets have been shown to polarize macrophages toward an M1 phenotype, augment dendritic cell maturation, and foster robust memory B-cell and antibody responses—key elements of long-term immunity against intracellular pathogens.

Altogether, NP-mediated approaches to intracellular infections provide a multifaceted strategy that combines precise drug delivery, host cell targeting, microenvironment-responsive release, and immune system engagement. These innovations represent a promising frontier in the treatment of persistent, drug-resistant, and immune-evasive bacterial infections.

### 5.6. Clinical Trials and Regulatory Perspectives

Despite promising preclinical outcomes, only a limited number of antimicrobial nanoparticle (NP) formulations have advanced to human clinical trials. One of the most notable examples is ARD-3150 (Pulmaquin^®^), an inhalable liposomal ciprofloxacin developed for patients with non-cystic fibrosis bronchiectasis chronically infected with *Pseudomonas aeruginosa*. In two Phase III trials—ORBIT-3 and ORBIT-4—Haworth et al. [158] reported that while ORBIT-4 showed a significant delay in the time to first pulmonary exacerbation, ORBIT-3 failed to meet its primary endpoint. When pooled, the trials did not yield statistically significant results, underscoring the translational gap between preclinical efficacy and consistent clinical outcomes. Another example includes PLGA-based nanocarriers loaded with colistin or meropenem, currently undergoing early-phase evaluation for treating MDR Gram-negative respiratory infections [159]. These biodegradable systems offer sustained pulmonary retention, reduced nephrotoxicity, and improved antimicrobial penetration, making them attractive candidates for future translation.

In contrast, AgNP-based wound dressings have already received regulatory approval and are widely utilized, particularly in European clinical settings. Products such as Acticoat™ and AgNP-containing hydrogels are regulated as medical devices rather than pharmaceuticals, benefiting from less stringent approval pathways. In a randomized controlled trial, Pathi et al. [160] compared Kadermin, a topical AgNP-based cream, with mupirocin in 86 patients with infected wounds. Kadermin achieved higher bacterial clearance (86 % vs. 65.1 % by day 5; *p* = 0.023) and significantly improved wound healing at day 28 (81.4 % vs. 37.2 %; *p* < 0.001) without reported adverse effects—highlighting its promise as an antibiotic-sparing therapy.

Regulatory frameworks are evolving to accommodate the complexities of nanomedicine. Vass et al. [161] described how the EMA has introduced innovation-enabling platforms such as the Innovation Task Force and the Quality Innovation Group to support the development of advanced therapeutics. Notably, nanotechnology-based antimicrobials were prioritized in the EMA’s Enabling Technologies Roadmap. Complementing these efforts, Emily et al. [162] discussed the U.S. FDA’s finalized guide “Drug Products, Including Biological Products, That Contain Nanomaterials,” which emphasizes defining critical quality attributes, rigorous physicochemical characterization, and reproducible manufacturing processes. These guidelines are pivotal in ensuring the safety, efficacy, and quality of nanoparticle-based therapeutics.

However, regulatory challenges remain. Soares et al. [30] highlighted the absence of standardized classification systems for nanomedicines, particularly in nanoparticle characterization and toxicology. They called for harmonized protocols that consider nanoparticle size, surface charge, and surface functionalization—critical determinants of in vivo behavior. Patra et al. [163] further noted that global health agencies are incorporating nanotechnology into their AMR strategies, supporting translational research and clinical validation of nano-enabled antimicrobials. Collectively, these efforts reflect a convergence in international regulatory thinking—aimed at aligning innovation with scalable, safe, and effective nanotherapeutic development.

Despite progress, several barriers continue to limit the clinical adoption of antimicrobial NPs. These include the lack of standardized toxicity assays, ambiguous classification (drug vs. device vs. combination), and manufacturing hurdles under good manufacturing practice conditions. Soares et al. [30] also noted batch-to-batch variability and long-term biosafety concerns, including potential nanoparticle-induced immunogenicity and microbiome disruption, as significant challenges.

To address these hurdles, global initiatives such as the EU Nanomedicine Characterization Laboratory and the FDA–EMA Nanomedicines Working Group are striving to harmonize preclinical testing and regulatory expectations. These collaborations aim to streamline approval pathways without compromising safety or efficacy. Advancing antimicrobial NP therapies will require early regulatory engagement, robust pharmacokinetic/pharmacodynamic modeling, scalable production, and comprehensive safety evaluations—including host–microbiota interaction studies. Continued investment in regulatory science and interdisciplinary collaboration is essential for realizing the clinical potential of nanomedicine.

**Figure 4:**
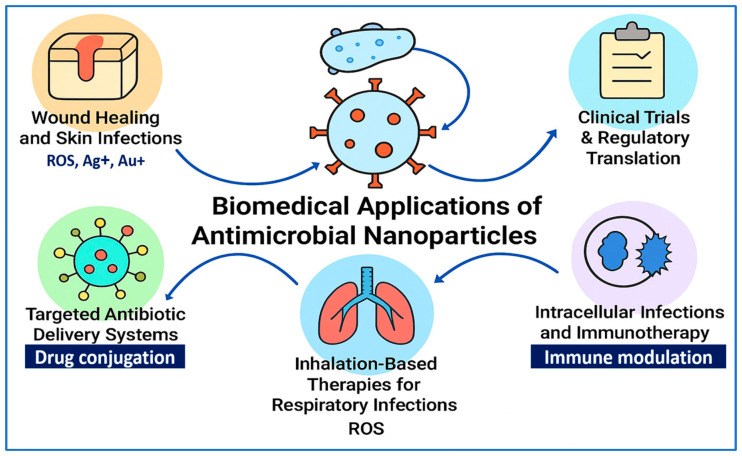
Biomedical applications of antimicrobial NPs. Key areas include wound healing, implant infection prevention, targeted drug delivery, inhalation therapies, intracellular infection treatment, and clinical/regulatory progress—underscoring their potential in combating resistant infections.

Figure 4 provides a visual summary of the key biomedical applications of antimicrobial NPs, illustrating their multifunctional roles in modern infection control strategies. These applications span six major areas: (1) wound healing and skin infections, where NPs such as Ag^+^, Au^+^, and ROS-generating agents facilitate antimicrobial action and tissue regeneration; (2) implantable device-related infections and clinical translation, emphasizing regulatory progress and therapeutic implementation; (3) targeted antibiotic delivery systems, leveraging nanoparticle–drug conjugation for enhanced site-specific therapy; (4) inhalation-based therapies for respiratory infections, utilizing NP-mediated ROS generation to eradicate pulmonary pathogens; (5) intracellular infections and immunotherapy, where immune-modulating NPs deliver antibiotics to phagocytic cells and stimulate host defense mechanisms; and (6) ongoing clinical trials and regulatory efforts, representing the pathway toward translational adoption. Together, these advances position antimicrobial NPs at the forefront of next-generation therapeutic interventions against multidrug-resistant and biofilm-associated infections.

### 5.7. Price and Affordability Constraints

Despite the therapeutic promise of antimicrobial NPs, their widespread clinical adoption is constrained by high production costs and scale-up limitations. Many synthesis processes—particularly those involving chemical or physical reduction methods—require expensive precursors, energy-intensive protocols, and sophisticated instrumentation, making them cost-prohibitive for large-scale pharmaceutical deployment. Sati et al. [164] emphasized that industrial-scale production of AgNPs remains economically challenging due to strict requirements for purity, particle uniformity, and environmental safety, which increase operational costs.

In response, green synthesis methods have gained traction as cost-effective and eco-friendly alternatives. By utilizing plant extracts and biological reducing agents, these approaches minimize reagent expenses and reduce environmental liabilities. Shahzadi et al. [41] demonstrated that biosynthetic routes offer a lower-cost, scalable pathway for AgNP production, particularly when optimized for plant metabolite content and reaction conditions. Similarly, Jebril et al. [165] reported that the use of multiple plant extract systems not only improved nanoparticle yield and stability but also significantly lowered overall synthesis costs, paving the way for broader clinical and industrial feasibility.

To facilitate equitable global access to nano-enabled antimicrobial therapies, future development must prioritize affordability alongside efficacy and safety. This includes standardizing green synthesis protocols, reducing batch-to-batch variability, and investing in scalable manufacturing platforms. Addressing cost barriers will be essential for integrating NPs into mainstream infection management strategies, particularly in low- and middle-income healthcare systems.

## 6. Translational Challenges and Safety Considerations of Antimicrobial NPs

Despite their therapeutic promise, antimicrobial NPs face numerous translational hurdles, including dose-dependent toxicity, immunogenicity, unpredictable biodistribution, manufacturing inconsistencies, and insufficient regulatory harmonization. Addressing these challenges through robust preclinical studies and standardized evaluation protocols is essential for their safe clinical implementation.

### 6.1. Toxicological Concerns and Dose-Dependent Toxicity

While metal-based NPs such as AgNPs and ZnO NPs demonstrate potent antimicrobial activity, they may also accumulate in organs and induce oxidative stress in vivo. In a good laboratory practice-compliant 90 day oral toxicity study in F344 rats, Kim et al. [166] reported that repeated exposure to ~56 nm AgNPs (30–500 mg/kg) led to dose-dependent silver accumulation in the liver and kidneys, bile duct hyperplasia, elevated liver enzymes, and weight loss—establishing a no observed adverse effect level (NOAEL) of ~30 mg/kg and a lowest observed adverse effect level (LOAEL) of ~ 125 mg/kg.

Cunningham et al. [167] showed that ultrasmall AgNPs (< 10 nm) induced more severe embryotoxicity in zebrafish, independent of silver ion release, emphasizing the size-dependent toxicity associated with nanoscale particles. Furthermore, Wen et al. [168] demonstrated that PEGylation of iron oxide NPs in rats significantly reduced their accumulation in the liver, spleen, and kidneys and attenuated inflammatory cytokine responses—highlighting the importance of surface modification in mitigating systemic toxicity.

### 6.2. Immune Activation and Inflammatory Risks

Cationic and lipid-based NPs—commonly employed in vaccines and antimicrobial formulations—can inadvertently activate innate immune pathways. Guo et al. [169] showed that intravenous administration of AgNPs in mice resulted in endothelial uptake, ROS overproduction, and disruption of VE–cadherin junctions, ultimately causing inflammation in the liver, lungs, and kidneys. Notably, the inflammatory response was size-dependent, with 75 nm and 110 nm AgNPs inducing more pronounced cellular infiltration than 10 nm particles.

Additionally, lipid-based NP components used in mRNA vaccine platforms have been implicated in immune activation. Connors et al. [170] found that empty lipid NPs enhanced dendritic cell maturation and cytokine production, with notable age-dependent effects in human peripheral blood cells. Complementary in vitro studies by Hanafy et al. [171] demonstrated that DOTAP-containing cationic lipids triggered IL-6 secretion and vascular permeability, consistent with the activation of the TLR/NLRP3 inflammasome pathways. Similarly, Lonez et al. [172] confirmed that ionizable cationic lipids can activate TLR2 and NLRP3 inflammasome signaling, resulting in IL-1β release—even in the absence of nucleic acids.

### 6.3. Protein Corona Formation and Biodistribution

When NPs encounter biological fluids such as blood or alveolar surfactant, they rapidly acquire a protein corona that reshapes their physicochemical identity. This layer significantly influences cellular uptake, organ distribution, clearance kinetics, and immunogenicity. Bai et al. [173] demonstrated that the in vivo protein corona—formed on engineered NPs following exposure to animal sera—profoundly altered biodistribution and toxicity profiles compared to in vitro predictions.

González-Vega et al. [174] further linked the composition of the protein corona to pulmonary deposition and inflammation. In rodent inhalation models, corona-coated AgNPs accumulated in alveolar macrophages and lung parenchyma, promoting neutrophil infiltration and cytokine elevation. Importantly, these effects varied depending on whether the NPs were inhaled as-is or pre-coated with coronas ex vivo.

### 6.4. Manufacturing, Scalability, and Standardization

The clinical scalability of antimicrobial NPs is hindered by batch-to-batch variability in synthesis, especially with green or hybrid methods. Small alterations in parameters such as pH, temperature, or extract concentration can result in significant differences in particle size, surface charge, stability, and antimicrobial efficacy. Liaqat et al. [175] compared various green synthesis protocols for AgNPs and found that these minor variations caused shifts in zeta potential (−15 mV to −5 mV), particle size (20–60 nm), and MIC values—emphasizing the need for standardization.

Herdiana et al. [176] demonstrated scale-up challenges using polymeric NP systems, where batch yield, monodispersity, and drug release profiles were inconsistent between lab-scale and continuous manufacturing platforms. To meet GMP requirements and ensure therapeutic reproducibility, manufacturing must define critical quality attributes, including polydispersity index, endotoxin levels, particle uniformity, and drug loading efficiency.

### 6.5. Green Synthesis and Biocompatibility

NPs synthesized via green chemistry—employing plant extracts or microbial agents—tend to exhibit enhanced biocompatibility while retaining antimicrobial efficacy. Liaqat et al. [175] tested over a dozen green-synthesized AgNPs and observed negligible cytotoxicity toward mammalian cells (e.g., fibroblasts, keratinocytes) at concentrations up to 50 µg/mL, with MICs ranging from 4 to 16 µg/mL. Naveen et al. [177] synthesized AgNPs using Aloe vera gel and found < 5 % hemolysis at 0.3 mg/mL, indicating excellent blood compatibility. Dudhagara et al. [178] reported similar results for lysozyme-stabilized AgNPs, which combined antibacterial and antiplatelet activity with minimal hemolytic effects (~ 6 %) at therapeutic doses.

### 6.6. PEGylation and Reduced Toxicity

Surface PEGylation improves NP safety by enhancing colloidal stability, reducing protein binding, and evading immune detection. Patlolla et al. [179] showed that oral administration of PEG-coated gold NPs in rats (12.5–100 µg/kg for 5 days) significantly lowered hepatic oxidative stress and histopathological alterations compared to uncoated controls. Cho et al. [180] found that intravenously administered PEGylated AuNPs (10 to 30 nm) circulated longer and accumulated less in the liver and spleen than their uncoated counterparts. These findings affirm PEGylation as a robust strategy for reducing toxicity while maintaining therapeutic efficacy.

### 6.7. Regulatory Frameworks and Clinical Translation

Clinical adoption of antimicrobial NPs is limited by fragmented regulatory guidelines. To date, only a few formulations—mainly topical AgNP-based gels—have entered early-phase trials (e.g., NCT03752424, NCT04894409, NCT04775238) [181]. Regulatory agencies like the FDA have issued generalized guidance on nanotechnology, but lack NP-specific criteria for pharmacokinetics, toxicity, and long-term safety assessments [182,183].

Kumari et al. [184] emphasized that divergent standards across regions complicate the translation of nanomedicine products. Nadar et al. [185] highlighted barriers to AgNP commercialization, including inconsistent classification as drugs or devices and a lack of standardized toxicity profiles. A 2024 review by Kumarasamy et al. [186] found that most clinical NP trials focus on antiviral or antifungal applications, with very few addressing antibacterial therapy—underscoring a translational gap.

A recent comparative regulatory review by Rodríguez-Gómez et al. [187] confirmed that both the EU and US frameworks still lack nanoparticle-specific approval pathways and exhibit inconsistencies in classification, safety testing requirements, and quality attribute definitions for nanotechnology-enabled health products. Likewise, Desai et al. [188] provide a global clinical perspective, identifying critical bottlenecks in pharmacokinetics characterization, immunotoxicity assessments, and standardization protocols—barriers that continue to slow the translation of nanoparticle therapeutics to human trials.

### 6.8. Microbiota–NP Interactions

Interactions between NPs and the host microbiota are increasingly recognized as critical to therapeutic safety and efficacy. Orally and intravenously administered antimicrobial NPs—particularly AgNPs, ZnO NPs, and TiO_2_ NPs—have been shown to induce gut dysbiosis and epithelial barrier disruption.

Van den Brule et al. [189] reported that AgNP exposure in mice led to reduced microbial diversity and a dose-dependent shift in the Firmicutes/Bacteroidetes ratio. Sulfidated AgNPs exhibited attenuated effects, suggesting environmental transformations may mitigate microbiota disruption. Lyu et al. [190] demonstrated that prenatal AgNP exposure caused long-term changes in microbiota composition and metabolic function in offspring, with implications for neurodevelopment and obesity.

Ren et al. [191] observed intestinal injury and leukocyte infiltration following AgNP exposure, while Wang et al. [192] noted decreased Gram-negative taxa and elevated serotonin levels in NP-treated mice—indicating potential neuroendocrine and metabolic consequences. Lamas et al. [193] provided a comprehensive mechanistic review showing that chronic dietary exposure to inorganic NPs—including Ag, TiO_2_, ZnO, and SiO_2_—frequently induces gut microbial imbalance, pro-inflammatory shifts (e.g., Proteobacteria overabundance), and reductions in beneficial taxa (e.g., *Lactobacillus*, *Bifidobacterium*), thereby promoting dysbiosis linked to metabolic disorders and inflammatory diseases.

Wu et al. [194] conducted a controlled rodent study demonstrating that oral TiO_2_ nanoparticle administration disrupted the gut bacterial community, reduced the abundance of short-chain fatty acid-producing probiotics, triggered intestinal inflammation and oxidative stress, and altered host lipid metabolism. An in vivo study by Kumar et al. [195] demonstrated that exposure to Ag, SiO_2_, and TiO_2_ nanoparticles in C57BL/6J mice induced dose-dependent alterations in gut microbiota composition, ranging from mild community shifts at lower doses to pronounced dysbiosis at higher exposures, characterized by elevated pro-inflammatory markers and reduced microbial diversity.

A recent study by Luo et al. [196] investigated the effects of dietary exposure to inorganic nanoparticles—including AgNPs, TiO_2_, and SiO_2_—on gut epithelial integrity in murine models. The findings revealed a dose-dependent downregulation of critical tight junction proteins (ZO-1, occludin, and claudin-1), along with mucosal thinning and increased intestinal permeability. These structural disruptions were accompanied by pronounced dysbiosis, characterized by significant reductions in beneficial genera such as Lactobacillus and Bifidobacterium, and elevated levels of colonic pro-inflammatory cytokines TNF-α and IL-6. Collectively, the results establish a mechanistic link between NP exposure, epithelial barrier impairment, intestinal inflammation, and microbial imbalance.

**Figure 5:**
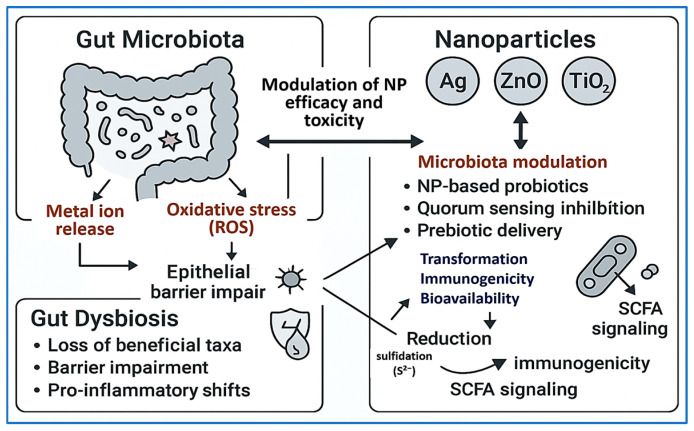
Gut microbiota–nanoparticle interplay. Antimicrobial NPs (Ag, ZnO, TiO_2_) disrupt gut homeostasis via ROS, metal ions, and barrier damage, while the microbiota modulates NP transformation, bioavailability, and immune effects—shaping both toxicity and therapeutic outcomes.

These emerging findings underscore the importance of incorporating microbiome-informed strategies into the development, safety evaluation, and regulatory assessment of antimicrobial nanotherapeutics—especially for formulations intended for oral or systemic administration. Figure 5 schematically illustrates the bidirectional interactions between antimicrobial nanoparticles and the gut microbiota, highlighting both microbiota-mediated NP transformation and NP-induced microbial and epithelial alterations.

## 7. Future Directions and Research Gaps

The advent of antimicrobial NPs has introduced transformative opportunities in combating MDR pathogens. However, several critical research gaps (Table 3) and translational challenges must be addressed to fully harness their clinical potential. A key area of innovation involves the development of stimuli-responsive or “smart” NPs that release their antimicrobial payloads selectively at infection sites—thereby minimizing systemic toxicity and enhancing therapeutic precision. For example, Li et al. [197] designed pH-sensitive PAMAM–HA–SNO “megamer” NPs co-loaded with nitric oxide and ciprofloxacin. These NPs disassembled in the acidic microenvironments characteristic of infected tissues, efficiently clearing *Staphylococcus aureus* infections in vivo while sparing healthy tissues. Similarly, Kalhapure et al. [198] engineered vancomycin-loaded solid lipid NPs that preferentially released the drug under acidic conditions in MRSA-infected murine skin, resulting in enhanced bacterial clearance and improved therapeutic outcomes.

Another prominent research frontier is biofilm eradication, which remains a major obstacle due to the protective nature of the extracellular polymeric matrix. Tasia et al. [199] developed DNase I-functionalized, silver-doped mesoporous silica NPs capable of enzymatically degrading extracellular DNA and delivering Ag^+^. These multifunctional NPs significantly disrupted both Gram-positive and Gram-negative biofilms in vitro. Other studies support the synergistic use of enzymatic disruption and targeted antimicrobial delivery to enhance biofilm penetration and eradication.

Despite growing preclinical evidence, the long-term biodistribution and toxicity of antimicrobial NPs remain under-investigated. Most studies focus on short-term efficacy, while chronic exposure—particularly in patients requiring repeated or prolonged therapy—raises safety concerns. Radiolabeled NP tracking in non-infected models has shown prolonged accumulation in the liver and spleen, emphasizing the need for long-term monitoring in relevant infection models. Future research should prioritize the development of advanced imaging protocols, pharmacokinetic modeling, and toxicity assays tailored to infectious disease contexts.

Environmental safety is another emerging concern as the widespread use of NPs in consumer and medical products may impact ecosystems and commensal microbial communities. Green synthesis approaches, such as those employing *Azadirachta indica* leaf extract, have produced AgNPs with potent antimicrobial activity and reduced toxicity toward soil and aquatic microbiota. These environmentally friendly methods warrant further exploration, and longitudinal environmental surveillance is essential to ensure sustainable deployment.

The integration of diagnostics and therapeutics in nanotheranostic platforms represents a promising avenue for personalized infection management. Hajfathalian et al. [200] developed dextran-coated gold-in-gold cage NPs capable of both photoacoustic imaging and photothermal ablation of biofilms in vivo, exemplifying dual-functionality systems. Likewise, enzyme-responsive fluorescent–antibiotic nanoshells offer real-time infection sensing with site-specific drug release [201]. These innovations align with precision medicine goals and may enable dynamic, patient-tailored interventions.

Lastly, international collaboration and regulatory harmonization are vital for advancing safe and effective clinical translation. Initiatives such as the EU’s Safe-by-Design framework and ISO/TR 10993-22 [202] guidelines for nanomaterial-containing medical devices are critical milestones. However, broader global adoption and cross-agency coordination remain urgently needed to accelerate clinical development, ensure safety, and streamline approval pathways.

**Table 3:** Key research gaps and future directions in antimicrobial NPs.

Research Gap	Description	Proposed Future Directions	Key References
Lack of stimuli-responsive drug delivery systems	Current NPs often release drugs passively, leading to off-target effects and reduced therapeutic precision.	Design infection-triggered or pH-/enzyme-responsive systems to enable site-specific activation.	[197]
Poor efficacy against biofilms	Biofilm matrices hinder NP penetration and antimicrobial activity, reducing treatment effectiveness.	Develop enzyme-functionalized NPs (e.g., DNase, protease) or ROS-generating NPs to disrupt biofilms.	[199,203]
Limited long-term safety and biodistribution data	Most studies assess short-term efficacy, lacking insight into chronic toxicity and organ accumulation.	Utilize radiolabeling or imaging-guided platforms to evaluate NP clearance, persistence, and tissue-specific effects over time.	[204,205]
Environmental impact and microbiome disruption	Persistent NPs, especially metal-based ones, may harm beneficial microbes in the environment or gut microbiota.	Prioritize green synthesis methods and biodegradable nanomaterials that limit ecological toxicity.	[206]
Fragmented regulatory frameworks	Lack of harmonized guidelines delays clinical translation and hinders global acceptance of NP therapeutics.	Develop unified regulatory protocols addressing NP characterization, manufacturing, and pharmacokinetics.	[29,207]
Insufficient integration with diagnostics (theranostics)	Few platforms allow simultaneous infection detection and treatment, limiting real-time precision therapy.	Create dual-function NPs combining antimicrobial delivery with imaging or biosensing elements.	[208]

## 8. Conclusions

NPs represent a powerful and versatile tool in the global effort to combat MDR pathogens. Their unique ability to disrupt bacterial membranes, penetrate biofilms, enable targeted drug delivery, and modulate host immune responses offers clear advantages over conventional antimicrobial therapies. Throughout this review, we have highlighted the broad spectrum of biomedical applications for NPs—ranging from wound healing and implant coatings to inhalation therapies and intracellular infection control—while emphasizing their mechanistic diversity and clinical potential. A particularly novel aspect addressed in this review is the bidirectional interaction between NPs and the host microbiota. While NPs provide enhanced antimicrobial efficacy, they also have the capacity to disrupt microbial homeostasis and influence host physiology, raising important concerns about long-term safety and unintended immunological consequences. These findings underscore the importance of considering not only efficacy but also the biological context in which NPs operate. As the field advances, it is imperative that future antimicrobial NP platforms embrace “safe-by-design” principles, integrating microbiota-informed approaches, precision pharmacokinetic profiling, and rigorous toxicological evaluation. Translating laboratory breakthroughs into clinical success will require robust regulatory frameworks, harmonized safety standards, and proactive engagement with regulatory agencies. Most importantly, progress in this domain will depend on interdisciplinary collaboration among microbiologists, nanotechnologists, clinicians, toxicologists, and policymakers. By aligning innovation with clinical and regulatory realities, antimicrobial NPs have the potential to transform the landscape of infectious disease prevention and treatment—ultimately serving as a cornerstone in the post-antibiotic era.

## Figures and Tables

**Table 1:** Comparative overview of recent metal and metal oxide NPs used for antimicrobial applications: synthesis methods, physicochemical characteristics, and functional outcomes.

Synthesis Method	Size (nm)	Surface Area (m^2^/g)	Temp/Time	Precursors	Morphology	Application	Reference
AgNPs							
Green synthesis using sumac (*Rhus coriaria*) extract	~4	41.8	25 °C/12 h	Sumac extract + silver nitrate (AgNO_3_)	Spherical, homogeneous distribution, face-centered cubic crystalline structure	Antibacterial activity against *B. cereus*, *B. subtilis*, *Enterococcus faecalis*, *P. aeruginosa*, and *C. albicans*; protection against plasmid DNA damage	[39]
Green synthesis using *Ocimum sanctum* (Tulsi) extract	~18–25	35.6	60–70 °C/2 h	AgNO_3_, aqueous tulsi leaf extract	Spherical (TEM, SEM)	Antibacterial and anticancer potential	[75]
Green synthesis using *Candida parapsilosis* (yeast) isolated from Sudanese soil	~10–25	48	Not specified	Yeast biomass extract + AgNO_3_	Spherical (HRTEM-confirmed)	Antibacterial against MDR bacteria; inhibition zones up to 29 mm; MIC/MBC 0.3125 mg/mL; synergistic antibiotic enhancement (up to 9.84-fold)	[76]
Chemical reduction optimized via Face-Centered Central Composite Design (FCCCD) with varying pH, AgNO_3_, sodium citrate (TSC), and NaBH_4_ concentrations	Majority < 10.30 nm (67.66 % of particles)	53	Ambient conditions/Reaction time not explicitly stated	AgNO_3_, TSC, NaBH_4_	Spherical and hemispherical	Antimicrobial and antifungal activity against *S. aureus, E. coli, E. coli* AmpC-resistant, and *C. albicans*; reduced cytotoxicity profile	[77]
Green synthesis using *Zingiber officinale* (ginger) extract	41.98	34.8	Ambient/Not specified	AgNO_3_, ginger extract (gingerol source)	Spherical	Antibacterial and antibiofilm activity against biofilm-associated *Enterococcus* spp. from urinary tract clinical isolates	[78]
**ZnO NPs**							
Sol–gel method	16–28	52.2	80 °C/4 h	Zinc acetate, NaOH	Hexagonal rods	Antibacterial textiles	[79]
Green synthesis (*Moringa oleifera* leaf extract)	55	15.43	~Room Temp/unspecified duration	Aqueous extract of *Moringa oleifera* leaves	Spherical	Hepatoprotective effect in CCl_4_-treated albino rats	[80]
Green synthesis (using *Scenedesmus obliquus* algae extract)	17–34	34.9	70 °C/2 h	Zinc nitrate hexahydrate, algae extract	Spherical	Phosphorus adsorption; antibacterial vs. *E. coli, S. aureus*	[81]
Green synthesis (*Allium cepa* (onion) extract)	8.13 (avg.)	33.3	60 °C/1 h (plus annealing at 400 °C for 2 h)	ZnSO_4_·7H_2_O, onion extract, NaOH	Spherical, aggregated (1–16 nm TEM)	Phosphorus adsorption (opt. at pH 3), antibacterial activity vs. *E. coli and S. aureus*	[82]
Green synthesis using *Trifolium pratense* (red clover) extract	20–80	14.29	60 °C/3 h (stirring) + drying and calcination at 400 °C	Zinc acetate dihydrate, red clover extract, NaOH	Spherical to irregular	Antibacterial activity against *E. coli*, *S. aureus*, and *C. albicans*	[83]
**CuO NPs/CuO/ZnO NPs**							
Green synthesis using *Syzygium aromaticum* (clove) extract	14.8	Not reported	80 °C/3 h (stirring) + drying at 100 °C, annealed at 400 °C	CuCl_2_·2H_2_O, clove extract	Spherical to aggregated	Structural and dielectric material development	[84]
Precipitation + polymer surface modification using PEG, PVP, and chitosan	13–50	Not reported	Room temp/24 h + drying at 60 °C	CuCl_2_·2H_2_O, NaOH, PEG/PVP/Chitosan (modifier)	Spherical and quasi-spherical	Antibacterial activity against *E. coli, S. aureus, B. subtilis, C. albicans*	[85]
Green synthesis using *Tribulus terrestris* leaf extract	18.9	Not re-ported	80 °C/2 h (stirring) + calcination at 500 °C	CuSO_4_·5H_2_O, plant extract	Spherical	Antimicrobial activity against *S. aureus, E. coli*; strong antioxidant activity (DPPH, ABTS)	[86]
Co-precipitation + multi-walled carbon nanotube (MWCNT) surface modification	13.44–21.96	33.83	70 °C/3 h + calcination at 400 °C	AgNO_3_, Cu(NO_3_)_2_·3H_2_O, Zn(NO_3_)_2_·6H_2_O, NaOH, MWCNT (5%)	Irregular, spherical, and semi-porous	Photocatalytic degradation of methylene blue (96.2 %) under sunlight; antibacterial activity vs. *S. aureus*, *E. coli*	[87]
Co-precipitation + annealing	15–30	41.7	500 °C/2 h	Cu(NO_3_)_2_, Zn(NO_3_)_2_, NH_4_OH	Aggregated clusters	Water disinfection	[88]
Physical vapor deposition (thermal evaporation) + post-annealing	~42 (CuO)/~39 (ZnO)	Not re-ported	Annealing at 350 °C for 1 h	High-purity Cu and Zn metals evaporated on glass substrate	Uniform thin film with granular nanostructure	Gas sensing (ammonia and acetone detection)	[89]
**TiO_2_ NPs**							
Sol–gel method	15–25	~112	80 °C/6 h	Titanium isopropoxide, ethanol, water, HCl	Spherical anatase	Antibacterial activity against *E. coli, S. aureus*, and *C. albicans* under UV illumination	[90]
Green synthesis using *Carica papaya* leaves	~50	78.9	Calcined at 400 °C/3 h	Titanium isopropoxide (TTIP), Carica papaya leaf extract	Spherical, smooth anatase NPs	Effective antibacterial activity against *E. coli, S. aureus*, and *B. subtilis*	[91]
Sol–gel (chemical)	~14–25	Not reported	Calcination at 400 °C	Titanium isopropoxide, ethanol	Spherical	Strong antibacterial activity against *E. coli* and *S. aureus*, and antifungal against *C. albicans*. Also effective against HSV-1 virus and in photocatalysis under UV light	[92]
Sol–gel synthesis (TiO_2_/Ag nanocomposite)	~10–20	Not specified	450 °C/2 h (calcination)	Titanium isopropoxide, AgNO_3_	Spherical with Ag clusters	Broad-spectrum antibacterial activity against *E. coli* and *S. aureus*	[93]
Sol–gel synthesis (commonly used in cited dental applications)	~15–30	Not reported	~400–500 °C (calcination)	Titanium isopropoxide, solvents	Spherical, anatase	Antibacterial coatings on dental implants, restorative materials, and endodontic sealers	[94]

**Table 2:** Comparison of metal and metal oxide NPs: target pathogens, MIC ranges, and biocompatibility profiles.

NP Type	Target Pathogens	MIC Range (µg/mL)	Cytotoxicity/Biocompatibility	Reference
AgNPs				
Actinobacteria-mediated, protein-capped	*E. coli*, *K. pneumoniae*, *P. aeruginosa*, *S. aureus*	8–128; 64–256	Dose-dependent cytotoxicity: IC_50_ (MTT) 16.3 µg/mL (RAW 264.7), 12.0 µg/mL (MCF-7); higher toxicity to cancer cells; ROS increase (MCF-7: 1.47–3.13×, RAW 264.7: 1.02–2.58×); LDH leakage up to ~45 %	[101]
*Salvia pratensis* aerial and root extracts	Broad-spectrum antibacterial and antifungal activity; strongest against *Penicillium* spp.	Bacteria: < 0.0039; Fungi (*Penicillium*): < 0.0391	Fully biocompatible with tested eukaryotic cells; no hemolysis at ≤150 µg/mL	[102]
Green-synthesized using *Achillea millefolium* extract	*E. coli, P. aeruginosa, S. aureus*, *C. albicans*	Bacteria: 3.12–25; Fungi: 6.25–50	No significant cytotoxicity against Vero and HaCaT cell lines at concentrations ≤100 µg/mL; >90% cell viability maintained	[103]
Green-synthesized using *Ocimum sanctum* leaf extract)	Not primarily antimicrobial-focused; study evaluated genotoxicity protection—AgNPs generally active against Gram-positive bacteria, Gram-negative bacteria, and fungi per the phytosynthetic AgNP literature	Not determined in this study	Showed protective effect against cyclophosphamide-induced DNA damage in human lymphocytes; no acute cytotoxicity reported in vitro	[104]
Green-synthesized using *Streptomyces antimycoticus* L-1	*S. aureus*, *B. subtilis*, *P. aeruginosa*, *E. coli*, *S. typhimurium*	6.25–100 ppm (zone of inhibition: 9.5–21.7 mm)	IC_50_ (Caco-2 cells) = 5.7 ± 0.2 ppm; 100 ppm considered safe for fabric coating; retained antibacterial activity after 10 wash cycles	[105]
**ZnO NPs**				
ZnO NPs immobilized in poly(allylamine hydrochloride)/alginate multilayers with vaterite on titanium coating	*S. aureus, S. epidermidis, C. albicans*	Not specified; >90% microbial viability reduction	Zn^2+^ release below cytotoxic limit for MC3T3-E1 preosteoblast cells; biocompatible for implant applications	[55]
Green-synthesized ZnO NPs using Casuarina equisetifolia leaf extract under UV-A and UV-C light	*B. subtilis*, *P. fluorescens*, *P. aeruginosa*	3.12–25	Reduced HepG2 cell viability to 36.97 %; highly biocompatible toward brine shrimp and human RBCs	[106]
Green-synthesized ZnO NPs using *Chelidonium majus* extract	*S. aureus (NCTC 4163, clinical), P. aeruginosa (NCTC 6749, clinical), E. coli (ATCC 25922, clinical), C. albicans (ATCC 10231, clinical), Aspergillus niger (ATCC 16404), Trichophyton rubrum (ATCC 28188)*	Bacteria: 3.12–25; Fungi: 6.25–50	Demonstrated high efficiency against human non-small cell lung cancer A549 cells; no specific normal cell cytotoxicity data reported in Abstract	[107]
ZnO-NPs reinforced in sodium alginate/hydroxyapatite scaffolds	*E. coli, S. aureus*	6.25–50	>70 % porosity; neutral pH; enhanced apatite deposition; high bioactivity; suitable for bone regeneration; no cytotoxicity reported	[108]
Green-synthesized ZnO nanoparticles (*Rosmarinus officinalis* L. extract; size 53–67 nm)	*S. aureus (Gram-positive), E. coli (Gram-negative)*	~81.4, 40.7, 20.35, and 10.17 µg/mL	Reported as biocompatible and non-toxic to humans in prior studies; no direct cytotoxicity assay performed in this study	[109]
**CuO NPs**				
Green-synthesized using *Trichoderma harzianum*; ~15–40 nm	*A. baumannii* clinical isolates	12.5–50	Low cytotoxicity toward human fibroblast cells at ≤50 µg/mL; also showed anticancer activity against HepG2 cells	[110]
Green-synthesized using metabolites of *Aspergillus niger* G3-1, spherical, 14–47.4 nm	*Sitophilus granarius, Rhyzopertha dominica*	50–100 ppm (dose-dependent mortality: 55–94.4 % for *S. granarius*, 70–90% for *R. dominica*)	Low phytotoxicity; 50 ppm promoted wheat growth, photosynthetic pigments, and antioxidant enzyme activity without affecting carbohydrate or protein content	[111]
Biosynthesized via *Aspergillus terreus* BR.1 from Allium sativum root	Different pathogenic bacteria and *Candida* species	25–50	Selective cytotoxicity: IC_50_ for MCF7 (159.2 µg/mL) and PC3 (116.2 µg/mL) cancer cells; higher IC_50_ for normal cells (Vero: 220.6 µg/mL, Wi38: 229.5 µg/mL); stable and non-toxic at lower concentrations	[112]
Biosynthesized via *Streptomyces rochei* cell-free filtrate	Multidrug-resistant *S. aureus,* including MRSA clinical isolates	6.5 (CuO-NPs, in combination with cefoxitin)	Non-toxic to human HFB-4 cells at ≤8 µg/mL (100 % viability); normal cell morphology observed	[113]
Mycosynthesized CuO-NPs	*P. aeruginosa*	MIC: 25 MBC: 50	Minimal toxicity toward normal human skin cells; selective cytotoxicity against HepG2 cancer cells; enhanced wound healing efficacy when combined with curcumin (CUR)	[114]
**TiO_2_ NPs**				
Aloe vera-mediated in PVA:SA nanocomposite films	*B. cereus, S. aureus, E. coli*	12.5–50	Biocompatible with human skin fibroblasts; suitable for wound dressing applications	[115]
Green-synthesized using *Withania somnifera* root extract	*E. coli, P. aeruginosa, MRSA, L. monocytogenes, Serratia marcescens, C. albicans*	6.25–50	Significant biofilm inhibition (43–71 % at 0.5× MIC for formation; 24–64 % for mature biofilms); cytotoxic against HepG2 liver cancer cells in vitro	[116]
TiO_2_ nano-colloids (sonochemically synthesized)	*P. aeruginosa, other Gram-positive and Gram-negative bacteria; Newcastle disease virus (NDV)*	2.24–21.21	Not specified; demonstrated antiviral and antibacterial activity at non-toxic doses in assays	[117]
Biosynthesized using *Streptomyces vinaceusdrappus* AMG31)	*Enterococcus faecalis, E. coli, Penicillium glabrum, Aspergillus niger, C. albicans*	Gram-positive: MIC 12.5; MBC 25; Gram-negative (*E. coli*): MIC 6.25; MBC 12.5	Minimal hemolysis (1.9 % at 1000 µg/mL); selective cytotoxicity toward cancer cells (Caco-2 IC_50_ 74.1 µg/mL, PANC-1 IC_50_ 71.04 µg/mL) with lower toxicity to normal WI38 cells (IC_50_ 153.1 µg/mL); hemocompatible and moderate wound healing effect	[118]
Vanillic acid–conjugated TiO_2_ NPs (VA–TiO_2_ NPs)	*S. aureus, Streptococcus mutans, Enterococcus faecalis, C. albicans*	60	Concentration-dependent apoptosis in human oral carcinoma KB cells at 15–120 µg/mL; promising for dental applications; not explicitly stated for normal cell safety	[119]

## Data Availability

Not applicable. No new data were created or analyzed in this study.

## Abbreviations

The following abbreviations are used in this manuscript:

**Abbreviation**	**Full Term**
AMR	Antimicrobial Resistance
NPs	Nanoparticles
MDR	Multidrug-resistant
AgNPs	Silver Nanoparticles
AI	Artificial Intelligence
AuNPs	Gold Nanoparticles
CFU	Colony-Forming Units
CuO	Copper Oxide
CuO NPs	Copper Oxide Nanoparticles
DNA	Deoxyribonucleic Acid
EMA	European Medicines Agency
FDA	U.S. Food and Drug Administration
GNS	Gold Nanostar
IFN-γ	Interferon Gamma
IL-1β	Interleukin-1 Beta
MIC	Minimum Inhibitory Concentration
Mo@ZIF-8	Molybdenum-Doped Zeolitic Imidazolate Framework-8
MOF	Metal–Organic Framework
MSNs	Mesoporous Silica Nanoparticles
NLCs	Nanostructured Lipid Carriers
NP(s)	Nanoparticle(s)
PEG	Polyethylene Glycol
PLGA	Poly(lactic-co-glycolic acid)
QS	Quorum Sensing
rGO	Reduced Graphene Oxide
RNA	Ribonucleic Acid
ROS	Reactive Oxygen Species
SCFAs	Short-Chain Fatty Acids
*S. aureus*	*Staphylococcus aureus*
*E. coli*	*Escherichia coli*
*P. aeruginosa*	*Pseudomonas aeruginosa*
*K. pneumoniae*	*Klebsiella pneumoniae*
*S. typhimurium*	*Salmonella typhimurium*
TiO_2_	Titanium Dioxide
TiO_2_ NPs	Titanium Dioxide Nanoparticles
TiN–Ag	Titanium Nitride–Silver
ZnO	Zinc Oxide
ZnO NPs	Zinc Oxide Nanoparticles
ZnO–Ag	Zinc Oxide–Silver
ZIF-8	Zeolitic Imidazolate Framework-8
